# Dermatophytosis in Iran: a sharp increase in cases caused by *Trichophyton mentagrophytes* var. *indotineae*

**DOI:** 10.3897/imafungus.17.180817

**Published:** 2026-02-19

**Authors:** Ameneh Takesh, Adéla Wennrich, Miroslav Kolařík, Ali Zarei-Mahmoudabadi, Neda Kiasat, Nader Pazyar, Abdollah Rafiei, Mahdi Abastabar, Iman Haghani, Mahboobeh Kharazi, Solmaz Basiri, Zahra Jahanshiri, Hossein Khodadadi, Rasoul Mohammadi, Hossein Zarrinfar, Zahra Seifi, Kambiz Diba, Hasti Kamali Sarvestani, Ali Rezaei-Matehkolaei

**Affiliations:** 1 Infectious and Tropical Diseases Research Center, Health Research Institute, Ahvaz Jundishapur University of Medical Sciences, Ahvaz, Iran Department of Mycology, Pasteur Institute of Iran Tehran Iran; 2 Laboratory of Fungal Genetics and Metabolism, Institute of Microbiology of the Czech Academy of Sciences, Prague, Czech Republic School of Public Health, Tehran University of Medical Sciences Tehran Iran; 3 Dermatology Department, School of Medicine, Ahvaz Jundishapur University of Medical Sciences, Ahvaz, Iran School of Medicine, Shiraz University of Medical Sciences Shiraz Iran; 4 Invasive Fungi Research Center, Communicable Diseases Institute, Mazandaran University of Medical Sciences, Sari, Iran Health Research Institute, Ahvaz Jundishapur University of Medical Sciences Ahvaz Iran; 5 Department of Medical Parasitology and Mycology, School of Medicine, Shiraz University of Medical Sciences, Shiraz, Iran School of Medicine, Ahvaz Jundishapur University of Medical Sciences Ahvaz Iran; 6 Department of Medical Parasitology and Mycology, Ardabil University of Medical Sciences, Ardabil, Iran Laboratory of Fungal Genetics and Metabolism, Institute of Microbiology of the Czech Academy of Sciences Prague Czech Republic; 7 Department of Mycology, Pasteur Institute of Iran, Tehran, Iran Communicable Diseases Institute, Mazandaran University of Medical Sciences Sari Iran; 8 Department of Medical Parasitology and Mycology, School of Medicine, Infectious Diseases and Tropical Medicine Research Center, Isfahan University of Medical Sciences, Isfahan, Iran School of Medicine, Urmia University of Medical Sciences Urmia Iran; 9 Allergy Research Center, Mashhad University of Medical Sciences, Mashhad, Iran Laboratory Sciences Research Center, Golestan University of Medical Sciences Gorgan Iran; 10 Laboratory Sciences Research Center, Golestan University of Medical Sciences, Gorgan, Iran Department of Medical Parasitology and Mycology, Ardabil University of Medical Sciences Ardabil Iran; 11 Department of Medical Parasitology and Mycology, School of Medicine, Urmia University of Medical Sciences, Urmia, Iran Allergy Research Center, Mashhad University of Medical Sciences Mashhad Iran; 12 Department of Medical Parasitology and Mycology, School of Public Health, Tehran University of Medical Sciences, Tehran, Iran School of Medicine, Infectious Diseases and Tropical Medicine Research Center, Isfahan University of Medical Sciences Isfahan Iran

**Keywords:** Antifungal resistance, dermatophytes, ITS genotyping, molecular epidemio­logy, recalcitrant dermatophytosis, squalene epoxidase, terbinafine resistance

## Abstract

Dermatophytosis is a common skin infection worldwide. In recent years, *Trichophyton mentagrophytes* var. indotineae has rapidly emerged as a leading cause of recalcitrant dermatophytosis. Iran shows high genetic diversity within the *T. mentagrophytes* complex, suggesting local emergence of new genotypes, yet nationwide data remain limited. We performed a one-year multicentre, hospital-based survey of referred cases (September 2023–September 2024) in nine provinces of Iran to describe the current epidemiology of dermatophytosis and to analyse mutations in the squalene epoxidase (SQLE) gene associated with terbinafine (TRB) resistance. Clinical samples were obtained from 2211 patients with suspected dermatophytosis. Dermatophytes were recovered from 1568 samples (71%). Species-specific PCR identified *T. mentagrophytes* var. *indotineae* in 1191 cases (76%), confirming it as the dominant agent across all clinical forms, age groups and provinces. The remaining isolates comprised other dermatophytes, mainly other members of the *T. mentagrophytes* complex, *T. tonsurans* and *Microsporum canis*. *Trichophyton mentagrophytes* var. *indotineae* caused most cases of tinea cruris, tinea corporis, mixed infections and generalised dermatophytosis. It also accounted for 59% of tinea unguium and 37% of tinea capitis cases. No significant sex bias was observed and most patients were 20–49 years old. The data did not indicate recent introduction of the infection from outside Iran, supporting ongoing local transmission. SQLE sequencing of 410 isolates revealed resistance-associated mutations in 45% of strains, mainly F397L (72%) and L393S (28%), with the highest frequencies in southern provinces. ITS sequencing of a subset of isolates confirmed their identity as *T. indotineae* and revealed two ITS genotypes, including a dominant genotype VIII and a new sub-genotype XXIX. These findings show that *T. indotineae* is now the dominant dermatophyte in Iran and carries a substantial burden of SQLE mutations, highlighting the urgent need for continued molecular surveillance and antifungal stewardship.

## Introduction

Dermatophytosis (tinea) is the most common fungal infection of keratinised tissues, including skin, hair and nails. Dermatophytes are widespread worldwide and are estimated to affect up to 25% of the global population. Although generally considered a minor health problem due to the availability of effective antifungal therapies, the overall burden remains substantial (Barac et al. 2024).

Since around 2016, several countries have reported extensive, recurrent and treatment‑refractory infections, characterised by widespread skin involvement, frequent relapses and poor response to standard therapy (Uhrlaß et al. 2022). The first large wave was described on the Indian subcontinent (Khurana et al. 2018; Ebert et al. 2020), followed by increasing reports from other parts of Asia, the Middle East and North Africa, Europe, Oceania and North America (Nofal et al. 2020; Nenoff et al. 2020; Brasch et al. 2021; Klinger et al. 2021; Pashootan et al. 2022; Astvad et al. 2022; Dellière et al. 2022; Jabet et al. 2022; Posso De Los Rios et al. 2022; Bortoluzzi et al. 2023; Bhuiyan et al. 2024; Ngo et al. 2024; Barac et al. 2024; Chua et al. 2025). On the Indian subcontinent, *T. rubrum* was historically the dominant cause of dermatophytosis (Kumar et al. 2023). In contrast, the recent increase in difficult-to-treat dermatophytosis has coincided with the emergence and increasing dominance of *T. mentagrophytes* var. *indotineae* (Ebert et al. 2020; Uhrlaß et al. 2022; Bhuiyan et al. 2024). This situation emphasises the need for systematic surveillance of dermatophyte species at regional and national levels.

A comparable shift has been observed in Iran, where recent field observations indicate that infections of glabrous skin have changed markedly in recent years. Atypical clinical forms, especially recalcitrant tinea cruris and tinea corporis with extensive skin involvement and repeated cycles of improvement and relapse, are now reported from different regions of the country and predominantly caused by the emerging lineage *T. mentagrophytes* var. *indotineae* (Taghipour et al. 2019, 2020; Batvandi et al. 2023; Haghani et al. 2024; Tamimi et al. 2024a, 2024b; Mirhendi et al. 2025). These chronic infections severely affect patients’ quality of life and are often associated with depression, sleep disturbances and social isolation. Together these findings show that the epidemiology of dermatophytosis in Iran has changed substantially following the emergence of this pathogen.

In 2021, this taxon was described as a distinct species, *Trichophyton indotineae*, based on ITS rDNA sequences and clinical features (Kano et al. 2021). It can also be reliably identified using MALDI‑TOF MS (Normand et al. 2022; de Paepe et al. 2024). Subsequent multilocus and phenotypic analyses suggested that it represents a single lineage within the complex, rather than a separate species (Tang et al. 2021). In line with its current treatment in internationally recognised taxonomic databases and supported by recent population‑genetic studies, we therefore adopt the designation *T. mentagrophytes* var. *indotineae* throughout this study (Švarcová et al. 2023, 2025).

From a clinical perspective, this variety causes extensive, inflamed and highly pruritic lesions that spread easily between individuals, particularly on the groin, trunk and extremities (Jabet et al. 2022; Uhrlaß et al. 2022). These infections are more difficult to treat than mild tinea of the nails or feet. Treatment failure is often linked to resistance to terbinafine (TRB), the drug of choice for *Trichophyton* infections (McClellan et al. 1999). Terbinafine resistance is caused by non-synonymous point mutations in the squalene epoxidase (SQLE) gene (Favre and Ryder 1996).

Following the first reports of epidemic infections caused by *T. mentagrophytes* var. *indotineae* on the Indian subcontinent, this pathogen has now been reported from all continents and the list of affected countries continues to increase (Gupta et al. 2025a). Since 2020, it has been also repeatedly detected in different regions of Iran (Taghipour et al. 2020; Batvandi et al. 2023; Haghani et al. 2024; Tamimi et al. 2024a, 2024b; Mirhendi et al. 2025). However, comprehensive national data on the epidemiology of these infections and the genetic basis of terbinafine (TRB) resistance in Iran are still lacking.

In this study, we analysed dermatophyte isolates from nine provinces of Iran using sequence-based methods and detection of TRB resistance. Our aim was to define the current epidemiological profile of dermatophytosis caused by the treatment-refractory pathogen *T. mentagrophytes* var. *indotineae* in Iran, alongside a brief comparison with data from other continents.

## Materials and methods

### Clinical sampling, patient data and ethical compliance

The study was conducted as a multicentre, hospital-based survey of referred cases over a one-year period from September 2023 to September 2024. During this time, dermatophyte isolates obtained from hair, nail and skin samples of patients referred to private mycology clinics and governmental university hospitals in nine provinces of Iran (Ardabil, Fars, Golestan, Isfahan, Khuzestan, Mazandaran, Razavi Khorasan, Tehran and West Azerbaijan) were included. As part of the routine diagnostic procedure, each clinical specimen was inoculated on to Sabouraud dextrose agar, supplemented with chloramphenicol and cycloheximide (Mycobiotic agar; Laboratorios Conda S.A., Madrid, Spain) and incubated in the dark at 25 °C.

Demographic data, type of tinea infection and, when available in medical records, history of travel abroad were recorded for all patients with confirmed dermatophytosis. All patients had at least one clinical sample (skin, hair or nail) positive for fungal elements by direct KOH microscopy. Written informed consent was obtained from all patients. The study was approved by the Research Ethics Committee of Ahvaz Jundishapur University of Medical Sciences (approval number IR.AJUMS.MEDICINE.REC.1401.085).

### Identification of clinical isolates

Identification of all isolates in this study was based on a set of DNA-based methods. Genomic DNA was extracted and purified from fresh colonies grown on Mycobiotic agar, as previously described by Liu et al. (2000). All isolates were first screened using a *T. mentagrophytes* var. *indotineae*–specific PCR with the primer pair C120-287F (5'-GGTCCGGAAGAGAGATCTCGC-3') and C120-287R (5'-CTACCTAGGTAGGTAGCTTGCTTATTG-3') (Batvandi et al. 2023). Isolates that were negative in the specific PCR were subsequently subjected to amplification of the complete ITS1–5.8S–ITS2 rDNA region using the universal fungal primers ITS1 (5'-TCCGTAGGTGAACCTTGCGG-3') and ITS4 (5'-TCCTCCGCTTATTGATATGC-3') (White et al. 1990). The PCR products were then digested with the restriction enzyme MvaI according to the manufacturer’s instructions (Thermo Fisher Scientific, Waltham, MA, USA).

Each PCR reaction was performed in a total volume of 50 μl containing 25 μl of 2× Taq DNA Polymerase Master Mix RED (Ampliqon-Biomol, Hamburg, Germany), 40 pmol of each forward and reverse primer, 100 ng of genomic DNA and nuclease-free water to volume. PCR amplification was carried out using a C1000 Touch™ Thermal Cycler (Bio-Rad Laboratories) with the following programme: initial denaturation at 95 °C for 5 min, followed by 35 cycles of denaturation at 95 °C for 30 s, annealing at 62 °C for 30 s and extension at 72 °C for 30 s, with a final extension at 72 °C for 10 min. For ITS rDNA amplification, the same cycling conditions were used, except that the annealing temperature was 58 °C and the extension time was 45 s.

For identification of non-*T. mentagrophytes* var. *indotineae* isolates, 10 μl of the digested ITS rDNA products were separated by electrophoresis on a 2% agarose gel. Fragment sizes were compared with reference patterns reported previously (Rezaei-Matehkolaei et al. 2013).

### ITS rDNA and Squalene epoxidase sequencing

To confirm the accuracy of the *T. mentagrophytes* var. *indotineae*–specific PCR, a total of 78 isolates were subjected to ITS rDNA sequencing using the primer pair ITS1F (5'-CTTGGTCATTTAGAGGAAGTAA-3') and ITS4 (5'-TCCTCCGCTTATTGATATGC-3') (Gardes and Bruns 1993). The isolates were selected randomly, with the aim of including representatives from all geographic regions. To assess the potential for terbinafine resistance amongst *T. mentagrophytes* var. *indotineae* isolates, partial sequencing of the SQLE gene was performed for 410 isolates. PCR amplification was carried out using the primers SQLE397S and SQLE397R, originally designed for detection of the Phe397Leu substitution in var. *indotineae* and related members of the *T. mentagrophytes/T. interdigitale* complex (Kano et al. 2021).

Each 20-µl reaction contained 1 µl of genomic DNA (50 ng ml^–1^), 0.3 µl of each primer (25 pM ml^–1^), 0.2 µl of MyTaq DNA polymerase and 4 µl of 5× MyTaq PCR buffer (Bioline, London, UK). Thermal cycling followed the manufacturer’s recommendations for MyTaq, with an annealing temperature of 55 °C. PCR products were purified enzymatically using ExoSAP as previously described (Werle et al. 1994). An ExoSAP working solution was prepared by a 1:9 dilution in nuclease-free H_2_O and added to the PCR products according to the manufacturer’s instructions, followed by incubation and enzyme inactivation. Purified amplicons were sequenced by Macrogen Sequencing Service (Amsterdam, The Netherlands).

Consensus sequences were generated from forward and reverse reads of both ITS and SQLE using MEGA 7. ITS sequences were compared using BLAST against the GenBank nucleotide database and the CBS database (Westerdijk Institute). Taxonomic assignment was based on comparison with the reference strain *T. mentagrophytes* var. *indotineae* NUBS19006 (GenBank LC508024) (Kano et al. 2021). To assess whether both detected ITS genotypes belonged to a single evolutionary lineage, a haplotype network analysis was performed using ITS sequences aligned with MAFFT v.7 (Katoh and Standley 2013). A TCS haplotype network was inferred in PopART (Leigh and Bryant 2015), inclu­ding reference sequences representing all ITS genotypes, defined by Uhrlaß et al. (2022). Sequences grouping with genotype VIII were assigned to *T. mentagrophytes* var. *indotineae* (Švarcová et al. 2023). Amino-acid sequences of SQLE were inferred by translating nucleotide sequences and compared with the reference sequence of the terbinafine-susceptible strain *T. mentagrophytes* TIMM2789 (GenBank KU242352) to identify mutation sites.

### Statistical analysis

Patient demographic data, including age, sex and place of residence, were entered into Microsoft Excel. Statistical analyses were performed using SPSS software version 21. The chi-square test was used to assess associations between dermatophyte species and demographic or geographic variables, with a significance level set at α = 0.05. For all chi‑square analyses, the null hypothesis stated that there was no association between species distribution and the variable of interest (age group, geographic region, clinical form or anatomical site) and the alternative hypothesis stated that an association was present.

## Results

**Figure 1.**
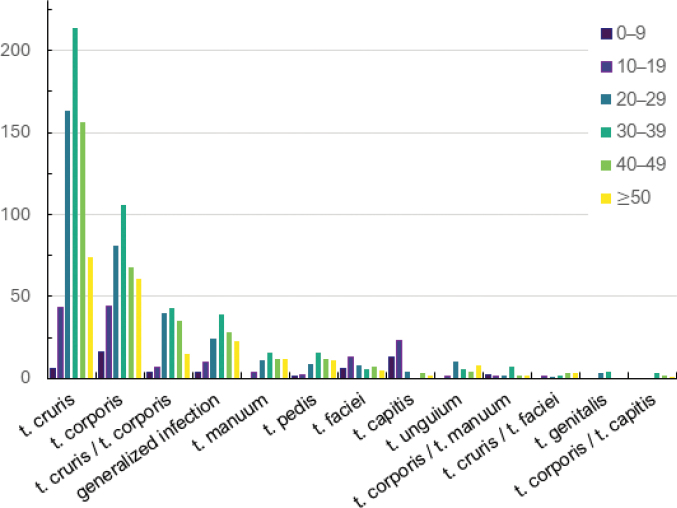
Distribution of *Trichophyton mentagrophytes* var. *indotineae* infections by clinical form and patient age. Adults aged 20–49 years represent the most affected group across major clinical presentations, particularly tinea cruris and tinea corporis. The association between age group and clinical form was statistically significant (χ^2^ test, *p* < 0.001).

In total, dermatophytes were cultured from clinical samples of 1568 out of 2211 suspected individuals (70.9%) (Tables 1, 2). The *T. mentagrophytes* var. *indotineae*–specific PCR was positive in 1191 cases (76%), confirming this taxon as the predominant etiological agent across all tinea types, provinces and age groups (Tables 1, 2, Fig. 1). The remaining 377 isolates consisted of members of the *T. mentagrophytes* species complex (TMSC), including genotype V (n = 44; 2.8%) and other TMSC genotypes (n = 161; 10.3%), as well as species outside the TMSC, namely *T. tonsurans* (n = 91; 5.8%), *Microsporum canis* (n = 42; 2.7%), *T. rubrum* (n = 17; 1.1%), *Epidermophyton floccosum* (n = 16; 1.1%), *T. violaceum* (n = 4; 0.3%) and *Nannizzia gypsea* (n = 2; 0.1%) (Tables 1, 2).

**Table 1.** Distribution of dermatophyte species by Iranian province and sex.

	*T. mentagrophytes*			*T. tonsurans*	*M. canis*	*T. rubrum*	*T. violaceum*	*E. floccosum*	*N. gypsea*	Total (%)
	var. *indotineae*	genotype V	others							
**Iranian province**										
Khuzestan	607	24	99	30	9	2	1	3	0	775 (49.4)
Fars	115	14	18	6	14	8	2	0	1	178 (11.4)
Mazandaran	130	2	6	5	1	0	0	0	0	144 (9.2)
Golestan	56	1	1	2	1	1	0	0	0	62 (4)
Isfahan	64	1	13	15	12	3	1	0	0	109 (7)
Ardabil	115	0	15	4	0	0	0	13	1	148 (9.4)
Tehran	75	2	3	26	3	3	0	0	0	112 (7.1)
Razavi Khorasan	19	0	3	3	2	0	0	0	0	27 (1.7)
West Azerbaijan	10	0	3	0	0	0	0	0	0	13 (0.8)
**Total**	1191	44	161	91	42	17	4	16	2	1568 (100)
**Sex**										
female	603	15	63	42	26	6	3	5	0	763 (48.7)
male	588	29	98	49	16	11	1	11	2	805 (51.3)
**Total**	1191	44	161	91	42	17	4	16	2	1568

**Table 2.** Distribution of dermatophyte species across clinical forms and age groups.

	*T. mentagrophytes* complex			*T. tonsurans*	*M. canis*	*T. rubrum*	*T. violaceum*	*E. floccosum*	*N. gypsea*	Total (%)
	var. *indotineae*	genotype V	others							
**Type of tinea**										
t. cruris	544	9	69	23	3	2	0	6	0	656 (41.8)
t. corporis	279	15	41	21	9	2	3	5	1	376 (23.9)
t. cruris / t. corporis	110	5	21	7	0	0	0	0	1	144 (9.2)
generalised infection	112	2	7	4	2	1	0	0	0	128 (8.2)
t. manuum	20	3	3	13	3	0	0	3	0	55 (3.5)
t. pedis	36	0	6	7	2	3	0	0	0	51 (3.3)
t. faciei	31	1	5	5	2	0	0	1	0	45 (2.9)
t. capitis	17	2	5	7	13	0	1	0	0	45 (2.9)
t. unguium	17	1	2	4	1	3	0	1	0	29 (1.8)
t. corporis / t. manuum	0	5	1	3	2	5	0	0	0	16 (1)
t. cruris / t. faciei	9	0	1	0	0	0	0	0	0	10 (0.6)
t. genitalis	6	0	0	0	0	1	0	0	0	7 (0.4)
t. corporis / t. capitis	0	1	0	0	5	0	0	0	0	6 (4)
**Total (%)**	1191 (76)	44 (2.8)	161 (10.3)	91 (5.8)	42 (2.7)	17 (1.1)	4 (0.3)	16 (1)	2 (0.1)	1568 (100)
**Age groups**										
0-9	23	6	3	6	9	1	3	1	0	52 (3.3)
10-19	105	5	13	15	9	1	0	1	0	149 (9.5)
20-29	275	7	40	17	6	2	0	7	2	356 (22.7)
30-39	358	5	55	29	7	5	0	3	0	462 (29.5)
40-49	260	17	30	15	6	2	0	2	0	332 (21.2)
≥ 50	170	4	20	9	5	6	1	**2**	0	217 (13.8)
**Total (%)**	1191 (76)	44 (2.8)	161 (10.3)	91 (5.8)	42 (2.7)	17 (1.1)	4 (0.3)	16 (1)	2 (0.1)	1568 (100)

Tinea cruris (n = 655; 41.8%), tinea corporis (n = 376; 23.9%), mixed tinea cruris/tinea corporis (n = 144; 9.2%), generalised infection (n = 128; 8.2%), tinea manuum (n = 55; 3.5%), tinea pedis (n = 51; 3.3%) and tinea capitis (n = 46; 2.9%) were the most frequently diagnosed clinical forms (Table 2). Remaining less frequent clinical forms are not listed here, but are included in Table 2. Specifically, *T. mentagrophytes* var. *indotineae* was predominantly isolated from patients with tinea cruris (n = 544; 45.7%), followed by tinea corporis (n = 279; 23.4%), generalised infection (n = 112; 9.4%), mixed tinea corporis and cruris (n = 110; 9.2%), tinea pedis (n = 36; 3%), tinea faciei (n = 31; 2.6%), tinea manuum (n = 30; 2.5%) and tinea unguium (n = 17; 1.4%) (Table 2).

The mean age of patients with *T. mentagrophytes* var. *indotineae* infection was 34.5 ± 13.6 years (median, 35; range, 1–85). The cohort included 588 males (49.4%) and 603 females (50.6%), with no significant difference in sex distribution (p = 0.664). Chi‑square tests were performed to evaluate whether species distribution differed across demographic and clinical categories. Chi-square analysis indicated significant associations between species distribution and age group, geographic region, clinical form of infection and involved body site (all p < 0.001), whereas no significant association was observed with sex (p = 0.664). Infections with *Trichophyton mentagrophytes* var. *indotineae* were significantly associated with southern provinces, particularly Khuzestan and Fars, were most prevalent in adults aged 20–49 years (Fig. 1) and were strongly linked to the clinical forms tinea cruris and tinea corporis.

Amongst the *T. mentagrophytes* var. *indotineae* infections with reliable patient history available (n = 73), none of the individuals had a documented history of travel abroad.

**Figure 2.**
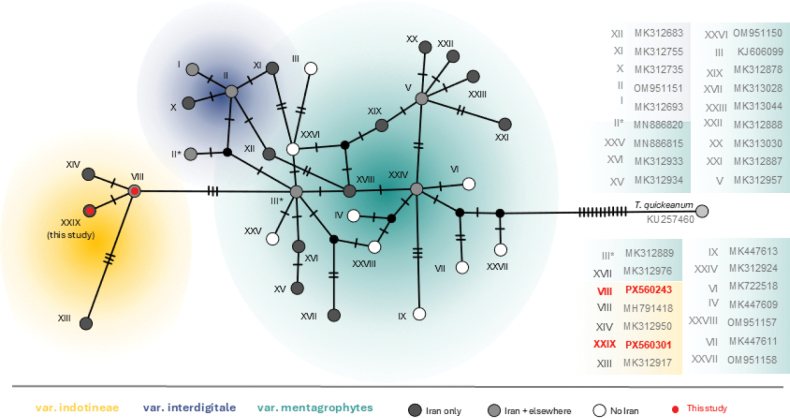
ITS rDNA TCS haplotype network (PopART) of the *Trichophyton mentagrophytes* species complex, including reference genotypes I–XXVIII (Uhrlaß et al. 2022) and sequences from this study (red). Most sequences belong to genotype VIII, while others represent a novel genotype XXIX within the *T. mentagrophytes* var. *indotineae* lineage. Dark and light grey circles indicate genotypes reported exclusively from Iran, reflecting high local diversity.

ITS rDNA sequencing confirmed all 78 isolates identified by taxa-specific primers as *T. mentagrophytes* var. *indotineae*, all clustering within the ITS genotype VIII lineage (Fig. 2). This set included two sub-genotypes within ITS genotype VIII (n = 57; accession numbers PX560243–PX560300) and a previously unrecognised ITS genotype within the *T. mentagrophytes* var. *indotineae* lineage XXIX (n = 20; accession numbers PX560301–PX560320).

SQLE mutations were detected in 184 of 410 isolates (44.8%) across all provinces. These mutations comprised three known point mutations resulting in two amino-acid substitutions: phenylalanine-to-leucine at position 397 (F397L; n = 132; 71.7%) and leucine-to-serine at position 393 (L393S; n = 52; 28.3%). Mutation frequencies varied geographically, with the highest proportions observed in Khuzestan (52.4%) and Fars (46%) and the lowest in Golestan (8.5%). New SQLE sequences have been deposited in GenBank under PX645601–PX645603.

In a pilot subset of sequenced isolates, ITS genotype VIII strains were either wild type or carried the F397L or L393S mutations, whereas isolates belonging to the newly-identified ITS genotype included one wild-type strain and 17 strains carrying the F397L mutation. The distribution and prevalence of these SQLE mutations in different populations will require further investigation.

## Discussion

This investigation represents the largest multicentre, hospital-based survey of referred dermatophytosis cases in Iran to date, focusing on infections caused by *Trichophyton mentagrophytes* var. *indotineae*. Before this recent epidemiological shift, dermatophytosis in Iran was primarily caused by non-*T. mentagrophytes* var. *indotineae* members of the *T. mentagrophytes* species complex, *T. rubrum*, *Microsporum canis*, *Epidermophyton floccosum* and *T. tonsurans* (Rezaei-Matehkolaei et al. 2013, 2016). Our data show that the epidemiological landscape of dermatophytosis has changed markedly within less than a decade, with *T. mentagrophytes* var. *indotineae* accounting for the highest proportion of cases amongst referred patients included in this survey (76%).

This finding is consistent with two recent regional studies from Tehran and Isfahan, in which *T. mentagrophytes* var. *indotineae* was by far the most common etiological agent of dermatophytosis, followed by *M. canis* and *T. tonsurans* as the next most prevalent species (Tamimi et al. 2024a; Mirhendi et al. 2025). Interestingly, our retrospective re-evaluation of ITS data reported by Taghipour et al. (2019) suggests that several *Trichophyton* isolates collected in 2011 and 2015 cluster within genotype VIII, which corresponds to what is now referred to as *T. mentagrophytes* var. *indotineae* in many recent studies. This indicates that the lineage was already present in Iran before the earliest reports from India, although these isolates had originally been identified as *T. interdigitale* under the former taxonomic framework (Taghipour et al. 2019).

The commonly proposed scenario that international travel and globalisation have driven the spread of *T. mentagrophytes* var. *indotineae* from the Indian subcontinent (Nenoff et al. 2019; Nenoff et al. 2020) may not fully explain the situation in Iran. In the study by Taghipour et al. (2019), none of the patients with available medical records reported travel to the Indian subcontinent or to MENA countries. Similarly, in our dataset of 73 confirmed cases, no history of international travel was documented. These findings suggest that the pathogen is established within Iran or that some lineages may have originated locally.

Taghipour et al. (2019) also reported striking genetic heterogeneity within the *T. mentagrophytes* complex in Iran, identifying 21 ITS genotypes, including *T. mentagrophytes* var. *indotineae*. Notably, twelve of these genotypes (Fig. 2) were newly described, indicating potentially unique local diversity. Consistent with this high genetic variability, we also identified a previously unrecognised ITS genotype XXIX within the *T. mentagrophytes* var. *indotineae* lineage in the present study (Fig. 2). Together, these findings raise the possibility that Iran could represent a centre of diversity for the *T. mentagrophytes* complex. Moreover, related lineages have been isolated from animal hosts, such as sheep, in rural regions of Iran, suggesting the presence of a local animal reservoir, although this hypothesis remains unconfirmed (Nikkholgh et al. 2023). The parallel increase in cases in Iran and India may reflect anthropogenisation of an originally zoophilic lineage; however, current data are insufficient to determine the origin or centre of diversity of *T. mentagrophytes* var. *indotineae*. Broader genomic and epidemiological studies will be required to resolve this.

Studies from India and Bangladesh have shown that *T. mentagrophytes* var. *indotineae* is now the dominant etiological agent of dermatophytosis, accounting for 93.5% and 96% of cases, respectively, with *T. rubrum* ranking as the second most common species (Ebert et al. 2020; Bhuiyan et al. 2024). In contrast, our comparative review of the literature (Table 3) indicates that, in Europe, North America, South America and Oceania, the epidemiology of dermatophytosis remains more heterogeneous. Although the emergence of this lineage has raised increasing concern in these regions, a substantial proportion of reported cases are linked to individuals originating from or travelling to endemic regions, including India, Bangladesh, Pakistan, Thailand, Myanmar and several MENA countries (e.g. Libya, Iraq, Bahrain and Egypt). Nonetheless, some reports indicate that infections can occur in individuals without a history of travel to endemic regions, suggesting the possibility of local transmission or introduction via secondary spread from non-endemic areas. Despite these emerging reports, *T. rubrum* continues to represent a major cause of dermatophytosis in many non‑endemic populations (Nenoff et al. 2020; Brasch et al. 2021; Klinger et al. 2021; Astvad et al. 2022; Dellière et al. 2022; Jabet et al. 2022; Posso-De Los Rios et al. 2022; Bortoluzzi et al. 2023; Cañete Gibas et al. 2023; Messina et al. 2023; Barnawi et al. 2025; Chua et al. 2025; de Almeida et al. 2025). In Denmark, surveillance data show that, although *T. mentagrophytes* var. *indotineae* has been detected, *T. rubrum* remained dominant, accounting for 81% of cases (Astvad et al. 2022). A similar pattern is observed in Australia, where *T. mentagrophytes* var. *indotineae* represented only 6.4% of dermatophyte isolates (Chua et al. 2025). In contrast, recent surveillance in the United Kingdom up to July 2024 documented a rapid increase, with *T. mentagrophytes* var. *indotineae* comprising 38% of dermatophyte isolates and potentially becoming the leading cause of tinea corporis (Abdolrasouli et al. 2025). Regional differences likely reflect a combination of travel-associated introductions, variation in healthcare access and surveillance intensity and the ability of the pathogen to exploit specific epidemiological niches.

**Table 3.** Reported cases of *Trichophyton mentagrophytes* var. *indotineae* infections from different countries.

Country	Predominant clinical form of infection	Predominant age range	Sex ratio (male/female)	Geographic origin of patients / travel history	SQLE amino-acid substitution(s)	Reference (year)
Greece	t. cruris / t. corporis	older adults (median age: 55)	(5/4)	data not available	F397L, L393S	Siopi et al. (2021)
France	t. corporis	20–57	(3/4)	India and Bangladesh	F397L, L393S, A448T	Dellière et al. (2022)
France	extensive dermatophytosis (not specified)	mean age: 30	6 (gender not specified)	Bangladesh, India and Myanmar	F397L, L393S, F397L/A448T, Ala448Thr	Jabet et al. (2022)
Germany	t. cruris / t. corporis, t. corporis, t. cruris	mean age: 26	(15/12)	German-born resident, India, Pakistan, Bangladesh, Libya, Iraq, Bahrain	F397L, F397L/A448T, L393F	Nenoff et al. (2020)
Germany	t. corporis / t. cruris	mean age: 29	(1/2)	India, Yemen	A448T	Brasch et al. (2021)
Denmark	NM	NM	NM	Denmark	F397L, L393F, F397L/A448T	Astvad et al. (2022)
India	t. corporis / t. cruris / t. faciei	mean age: 28	(138/63)	India	F397L, F397L/A448T, L393F, H440T, S443P, L335F, S395P, A448T	Ebert et al. (2020)
India	t. corporis / t. cruris	mean age: 34.9	(44/20)	India	F397L, L393F	Khurana et al. (2018)
USA/Canada (North America)	t. corporis / t. cruris	adults	NM	multiple U.S. states + Canada (North American surveillance)	F397L*, L393F*	Cañete-Gibas et al. (2023)
Canada (Ontario)	t. corporis / t. cruris	20–64	~1.0	Ontario, Canada; mainly imported but possible local transmission	F397L, L393F/S	McTaggart et al. (2024)
Canada	t. corporis / t. cruris	mean age: 42.75	(4/4)	All immigrated from India	NM	Posso-De Los Rios et al. (2022)
Brazil (South America)	t. cruris / t. corporis	adults	NM	São Paulo, Brazil (travel history to USA/Europe)	NM	de Almeida et al. (2025)
Argentina (South America)	t. corporis	21 years	female	Argentina (travel history to Mexico)	NM	Messina et al. (2023)
Italy	t. cruris / t. corporis	> 18 years	(4/1)	Italy, Pakistan, Egypt	F397L, , L393S, F415C	Bortoluzzi et al. (2023)
Bangladesh	t. cruris / t. corporis / t. genitalis	21-50	(60/36)	Bangladesh	F397L, L393S, A448T, S436A, F397I, L393F, F397L/A448T, N429D (Asn429Asp)	Bhuiyan et al. (2024)
Switzerland	t. cruris / t. corporis, multiple areas	median age: 32	(5/7)	India, Bangladesh, Thailand	F397L	Klinger et al. (2021)
Australia	t. cruris / t. corporis	median age: 31.3	(5/6)	NM	NM	Chua et al. (2025)
Vietnam	t. corporis / t. cruris	23-60	(3/1)	Vietnam, India	F397L/Q417H/D460G, Q417H/D460G	Ngo et al. (2024)
Iraq	t. corporis / t. capitis	≥20	(38/14)	NM	F397L, F397L/F311L	Mahmood et al. (2024)
Iran	t. cruris / t. corporis / t. pedis	31–50	(6/4)	Iran	F397L	Pashootan et al. (2022)
Iran	t. corporis / t. cruris	mean age: 33.6	(16/24)	Iran	F397L/A448T, F397L, L393S	Haghani et al. (2024)
Iran	t. cruris / t. corporis	21–30	(87/72)	Iran	NM	Mirhendi et al. (2025)
Iran	t. cruris / t. corporis, generalised infection	> 20 years	(588/603)	Iran	F397L, L393S	Current study

t., tinea; NM, not mentioned

For many years, non-*T. indotineae* members of the *T. mentagrophytes* species complex, together with *T. rubrum*, *Epidermophyton floccosum*, *Microsporum canis* and *T. tonsurans*, were the main causative agents of dermatophytosis in Iran, particularly in cases of tinea pedis, tinea unguium and tinea corporis (Rezaei-Matehkolaei et al. 2013, 2016). Based on all available evidence, including the findings of the present survey (Table 1), the epidemiological situation in Iran now closely resembles that of the Indian subcontinent, with *T. mentagrophytes* var. *indotineae* rapidly replacing other species as the dominant pathogen. In terms of incidence, our results are also comparable with those from the neighbouring country Iraq, where *T. mentagrophytes* var. *indotineae* was identified as the leading dermatophyte causing infections in Al Diwaniyah (Mahmood et al. 2024).

Our robust data from Iran show that *T. mentagrophytes* var. *indotineae* accounted for the majority of tinea cruris (544/655; 83%), tinea corporis (279/376; 74.2%), generalised infections (112/128; 87.5%) and mixed tinea corporis/cruris (110/144; 76%) (Table 2). This pattern primarily reflects the ecological niche and transmission dynamics of this lineage, which predominates in infections of the groin and trunk and is facilitated by close contact, shared environments and behavioural factors. These proportions are consistent with reports from recent studies in Asia and Europe, where *T. mentagrophytes* var. *indotineae* was responsible for 15–82% of tinea cruris and 38–100% of tinea corporis cases (Khurana et al. 2018; Ebert et al. 2020; Nenoff et al. 2020; Dellière et al. 2022; Tamimi et al. 2024a; Mirhendi et al. 2025). This clinical pattern does not fundamentally differ from infections caused by other *T. mentagrophytes* lineages, as groin and trunk involvement is common across dermatophyte species. In our dataset, the high proportion of these forms primarily reflects the regional dominance of *T. mentagrophytes* var. *indotineae* (see Table 2; Nenoff et al. (2020)).

Until about a decade ago, *E. floccosum* and *T. rubrum* were by far the most common causes of tinea cruris in Iran (Rezaei-Matehkolaei et al. 2013, 2016). In 2025, amongst 655 isolates from tinea cruris cases across nine provinces (Table 1), only 16 were identified as *E. floccosum* (Ardabil, n = 13; Khuzestan, n = 3; other provinces, n = 0), indicating a marked nationwide decline of this species. By comparison, two studies from southwest Iran conducted during 2012–2014 reported *E. floccosum* in 74% (38/51) and 83% (192/231) of groin infections, respectively (Ansari et al. 2016; Rezaei-Matehkolaei et al. 2016). Recent studies from Iran consistently show that the majority of dermatophytoses — particularly tinea cruris and tinea corporis — are now caused by *T. mentagrophytes* var. *indotineae* (Tamimi et al. 2024a; Mirhendi et al. 2025).

Although strict anatomical site preference has not been demonstrated for *T. mentagrophytes* var. *indotineae*, its high prevalence in endemic regions is reflected in a predominance of groin, trunk and mixed-skin infections. In this context, we also observed seven cases of tinea genitalis with isolated genital involvement, all caused by this lineage, suggesting that sexual transmission — previously documented for *T. mentagrophytes* type VII — may also occur in *T. mentagrophytes* var. *indotineae* (Descalzo et al. 2025). The true frequency is likely underestimated, as some cases, classified as tinea cruris, may be linked to sexual contact, but exposure history is often difficult to document.

Beyond glabrous skin, our data show that *T. mentagrophytes* var. *indotineae* is now detected across a broad range of anatomical sites, including the scalp and nails, where infections are classically attributed to *M. canis*, *T. tonsurans* or *T. rubrum*. This contrasts with earlier reports from Europe, Australia and Canada, as well as with smaller recent Iranian studies, in which nail and scalp involvement by this lineage was rare or absent (Rezaei‑Matehkolaei et al. 2013; Rezaei‑Matehkolaei et al. 2016; Klinger et al. 2021; Posso‑De Los Rios et al. 2022; Dellière et al. 2022; Bortoluzzi et al. 2023; Barac et al. 2024; Tamimi et al. 2024a; Mirhendi et al. 2025; Chua et al. 2025).

Earlier Iranian surveys reported tinea unguium in 21.3% and 6.9% of dermatophytosis cases, caused exclusively by *T. rubrum*, *T. interdigitale* and *E. floccosum* (Rezaei-Matehkolaei et al. 2013, 2016). In contrast, in our nationwide study, tinea unguium accounted for only 1.8% of all cases (29/1568) and 58.6% of nail infections were caused by *T. mentagrophytes* var. *indotineae*. A similar pattern was observed for tinea capitis, where *T. mentagrophytes* var. *indotineae* was the leading agent (37%), followed by *M. canis* and *T. tonsurans*, consistent with recent data from Iraq (Mahmood et al. 2024). Together, these findings indicate that *T. mentagrophytes* var. *indotineae* is now involved not only in infections of glabrous skin, but also of the scalp and nails.

Regarding sex distribution, no statistically significant difference was detected amongst patients infected with *T. mentagrophytes* var. *indotineae* (Table 1). In contrast, some studies from France (Dellière et al. 2022), Germany (Brasch et al. 2021), Switzerland (Klinger et al. 2021) and Australia (Chua et al. 2025) have reported a higher frequency of infections in females, whereas, reports from India, Bangladesh and Iraq showed approximately twice as many infections in males (Khurana et al. 2018; Ebert et al. 2020; Bhuiyan et al. 2024; Mahmood et al. 2024). Given that many published studies are based on case reports or small case series, it is still too early to draw firm conclusions about a true sex predisposition.

With respect to age, *T. mentagrophytes* var. *indotineae* infections occurred mainly in adults, particularly in the age groups 20–29 to 40–49 years (Table 2, Fig. 1). This is consistent with published data (Table 3), showing that children are less frequently affected and that the infection predominantly involves older adolescents and adults, with reported mean ages above 26 years (Posso-De Los Rios et al. 2022). Higher social and occupational mobility in adults likely increases exposure through shared environments, such as workplaces, gyms, sports facilities and public transportation. Intimate contact may also contribute, as tinea cruris and tinea genitalis can be transmitted sexually, corresponding to peak sexual activity in these age groups (Gupta et al. 2025b). In addition, behavioural and environmental factors, such as genital shaving or waxing, commonly practised for cosmetic or hygiene purposes, have been proposed as additional risk factors for acquisition of dermatophyte infections in adults, whereas children have lower exposure to these factors (Descalzo et al. 2025).

Regarding SQLE profiling, only two point mutations — F397L (71.7%) and L393S (28.3%) — were detected amongst the Iranian *T. mentagrophytes* var. *indotineae* isolates analysed in this study. Both mutations have previously been linked to terbinafine resistance in numerous Asian and European isolates (Table 3). Globally, F397L is reported as the most frequent SQLE mutation (33.0%), followed by Ala448Thr (A448T; 24.5%) and the combined F397L + A448T mutation (18.9%), whereas L393S accounts for 13.7% and S436A for 2.4% of strains (Gupta et al. 2025a).

In three earlier studies from Iran, F397L — either alone or in combination with A448T — was likewise the dominant mutation associated with terbinafine resistance (Taghipour et al. 2020; Pashootan et al. 2022; Haghani et al. 2024). In the present study, we used the primer pair introduced by Kano et al. (2021) to partially sequence an approximately 400-bp region of the SQLE gene, specifically targeting mutations at positions 393 and 397. Consequently, additional mutations such as A448T, which are not directly associated with clinical or in vitro terbinafine resistance, may not have been detected (Taghipour et al. 2020; Gupta et al. 2025a).

Previous genetic surveys from Iran reported SQLE mutation rates of 17.8% in 2020 (Taghipour et al. 2020) and 14.35% in 2024 (Haghani et al. 2024). In contrast, the mutation rate in the current study increased to 44.8% (184 of 410 sequenced isolates), which is of serious concern. Based on all available data from Iran, F397L and L393S appear to be the predominant SQLE mutations associated with likely terbinafine resistance in *T. mentagrophytes* var. *indotineae* across the country. However, because antifungal susceptibility testing was not performed for the sequenced isolates, direct genotype–phenotype correlations could not be established in this study. The emergence of terbinafine-resistant *T. mentagrophytes* var. *indotineae* has important clinical implications. Guidance documents stress accurate species identification, awareness of resistance and appropriate management of recalcitrant dermatophytosis (Drake et al. 1996; Rajagopalan et al. 2018). Recent diagnostic guidance highlights the importance of differentiating terbinafine-resistant strains, frequently associated with lineages commonly referred to as *T. mentagrophytes* var. *indotineae*, from other dermatophytes using a combination of molecular and phenotypic approaches (Al-Janabi 2025). These recommendations are supported by our findings, particularly by the high prevalence of SQLE mutations observed amongst Iranian isolates.

## Conclusions

In summary, this nationwide survey demonstrates that *Trichophyton mentagrophytes* var. *indotineae* has rapidly become the leading cause of dermatophytosis in Iran, largely replacing historical etiological agents, such as *T. rubrum*, *E. floccosum*, *T. tonsurans* and other members of the *T. mentagrophytes* species complex. This lineage now dominates not only tinea cruris and tinea corporis, but also contributes substantially to generalised infections, tinea unguium and tinea capitis, indicating broad anatomical tropism and a strong competitive capacity across skin, hair and nails. The absence of documented international travel in our cohort, together with previously reported local genetic diversity, suggests that *T. mentagrophytes* var. *indotineae* is now firmly established within Iran, although its ultimate geographic origin and evolutionary history remain unresolved.

The high and increasing proportion of isolates carrying the SQLE mutations F397L and L393S — both linked to terbinafine resistance — indicates a growing reservoir of likely resistant strains. Although antifungal susceptibility testing was not performed, the marked rise in mutation frequency compared with earlier Iranian studies is clinically concerning and points to a substantial risk of future treatment failures. These findings highlight the urgent need for routine molecular identification and SQLE genotyping, coupled with strengthened national surveillance. Broader genomic studies, targeted investigation of potential animal reservoirs, and integration of clinical outcome data will be essential to clarify the origin and transmission dynamics of these lineages and to guide effective therapeutic strategies.

## Additional information

### Use of AI

Artificial intelligence was used solely as a language-editing support tool during the preparation of this manuscript. It assisted in improving grammar, clarity, and stylistic consistency of the text written by the authors. All scientific content, data analysis, interpretations, and conclusions were entirely generated, verified, and approved by the authors.

### Adherence to national and international regulations

All the fungal strains used in this study have been legally obtained, respecting the Convention on Biological Diversity (Rio Convention).

### Author ORCIDs

Adéla Wennrich https://orcid.org/0000-0002-7986-6688

Miroslav Kolařík https://orcid.org/0000-0003-4016-0335

Ali Zarei-Mahmoudabadi https://orcid.org/0000-0002-5347-2082

Neda Kiasat https://orcid.org/0000-0001-8658-602X

Nader Pazyar https://orcid.org/0000-0002-7069-2255

Abdollah Rafiei https://orcid.org/0000-0001-8997-9056

Mahdi Abastabar https://orcid.org/0000-0002-0016-2849

Iman Haghani https://orcid.org/0000-0001-9723-7618

Zahra Jahanshiri https://orcid.org/0000-0001-9431-9335

Hossein Khodadadi https://orcid.org/0000-0001-9507-8764

Rasoul Mohammadi https://orcid.org/0000-0002-8220-4511

Hossein Zarrinfar https://orcid.org/0000-0002-1449-4668

Zahra Seifi https://orcid.org/0000-0002-9507-2773

Kambiz Diba https://orcid.org/0000-0001-8401-5282

Hasti Kamali Sarvestani https://orcid.org/0000-0002-5431-5468

Ali Rezaei-Matehkolaei https://orcid.org/0000-0002-3119-8342

### Data availability

The ITS rDNA sequences generated in this study have been deposited in the GenBank database under accession numbers PX560243–PX560320. The SQLE sequences obtained in this study are available in GenBank under accession numbers PX645601–PX645603. Due to ethical restrictions, individual-level clinical metadata are not publicly available, but can be accessed from the corresponding author upon reasonable request.
